# Transmission of yeast and bacterial symbionts between sexual partners in *Drosophila suzukii* and *Drosophila melanogaster*

**DOI:** 10.1098/rsos.241149

**Published:** 2025-02-19

**Authors:** Robin Guilhot, Anne Xuéreb, Simon Fellous

**Affiliations:** ^1^ CBGP, Univ Montpellier, CIRAD, INRAE, Institut Agro, IRD, Montpellier, France

**Keywords:** sexual transmission, *Drosophila suzukii*, *Drosophila melanogaster*, extracellular symbionts, sterile insect technique

## Abstract

Sexually transmitted symbionts can substantially affect the performance and evolution of their hosts. From a pest control perspective, the sexually transmitted microorganisms of insects can be considered powerful biological control agents or probiotics. The sterile insect technique (SIT) is currently being developed as a new tool to control *Drosophila suzukii*, a major crop pest. With considerable numbers of mass-reared insects released to mate with wild individuals, understanding how microbiota transfers between adult insects is necessary not only to improve the effectiveness of the technique but also to prevent the potential spread of non-native and harmful microorganisms in wild arthropod populations and their environment. We investigated the sexual transmission of yeast and bacterial symbionts in *Drosophila suzukii* and in the universal model *Drosophila melanogaster*. In an ecologically realistic set-up, we combined behavioural and microbiological measurements using flies associated with four microorganisms. We detected microbial transmission more frequently in mated flies, which was mostly influenced by the identity and density of microbial strains in the donor and recipient hosts. Our results suggest the importance of using hosts associated with several microorganisms in microbiota transmission studies, open new perspectives for crop protection and point to an overlooked non-target effect of the SIT.

## 1. Introduction

Adult arthropods are associated with extracellular microbial symbionts that can be inherited from previous life stages as well as acquired from the environment [1]. Microbial symbionts can notably be transferred between congeners when they share habitats or feed on the same resource [2,3] and through sexual behaviour, during courtship and mating [4,5]. Sexual transmission of microbial symbionts has been reported in several arthropods [3,6–11]. As for other modes of transmission, sexual transmission of a microbial symbiont affects the ecology and evolution of the host–symbiont interactions [12–14]. Although sexually transmitted diseases or other pathogens negatively affect their hosts, transfers of microbial symbionts between sexual partners can also reduce chances of reproductive parasitism, i.e. conflict between symbiont and host genes [15]. Sexual transmission of microbial symbionts can also facilitate host associations with beneficial symbionts by increasing the symbiotic diversity of the sexual partners [8,14]. More generally, the ecological and evolutionary trajectories of holobionts, i.e. assemblages of hosts and their symbionts, depend on the transmission dynamics of these symbionts. Understanding microbiota assembly in model organisms is therefore of key importance. When live microorganisms are considered as potential biological control agents [16] or probiotics [17,18] for pest control, the ecological and demographic consequences of releasing such microorganisms for target populations have to be evaluated, including through the spectrum of sexual transmission. Similarly, when mass-bred insects are released during sterile insect technique (SIT) programmes, sexual transmission of symbionts may lead to the colonization of wild populations with microorganisms from sterile insects. It is therefore necessary to understand sexual transmission of microbiota members for fundamental and applied purposes.

The genus *Drosophila* comprises the universal model insect *Drosophila melanogaster* and the fruit pest *Drosophila suzukii. D. melanogaster* is widely used to understand the symbiosis and the role of the microbiota in physiology, ecology and evolution [19,20], while *Drosophila suzukii* is a major soft and stone fruit pest that recently invaded large parts of the world [21,22]. Unlike most other *Drosophila* flies, *D. suzukii* females oviposit on ripening, undamaged fruits, in which larval damage leads to critical yield losses [23]. In the wild, interactions between *D. melanogaster* and *D. suzukii* flies [24–26] are widely mediated by their microbiota, illustrating the importance and potential of extracellular symbionts for pest management [27]. As SIT is currently developed for *D. suzukii* control [28–30], it is necessary to study the sexual transmission of *D. suzukii* microbiota members to understand its implications for wild populations and identify opportunities for technical improvement. The transmission of extracellular microorganisms during mating was reported in *Drosophila* species including *D. melanogaster* [1,5], however, under very artificial conditions. Here, we investigated the sexual transmission of microbiota members in *D. melanogaster* and *D. suzukii* in an ecologically realistic set-up. To this aim, we combined sexual behaviour observations and microbiology measurements using gnotobiotic flies and natural fruit. We first studied how mating affected the transmission of yeasts and bacteria between *Drosophila* males and females. Second, we focused on mated flies and analysed the factors that influenced microbial transmission such as microbial identity and density.

## 2. Material and methods

### 2.1. *Drosophila* rearing

We used two fly colonies: a *D. suzukii* population founded from wild adults collected in 2013 in Gaujac (Occitanie, France) and maintained in the laboratory for 90 generations, and a *D. melanogaster* population founded from adults collected in 2017 from wild pomegranate in Montpellier (Occitanie, France) and maintained in the laboratory for 40 generations. We maintained these colonies at 21°C with 70% humidity and a 14 h photoperiod on artificial diet (37.5 g l^−1^ dried carrot powder, 37.5 g l^−1^ sugar, 22.5 g l^−1^ inactive dry yeast, 15 g l^−1^ corn meal, 11.25 g l^−1^ agar, 5 ml l^−1^ propionic acid, 3.3 g l^−1^ nipagin in 2.5 ml l^−1^ ethanol).

### 2.2. Microbial isolates

We used two different yeasts and two different bacteria commonly found associated with *Drosophila* flies: the yeast *Hanseniaspora uvarum* (isolated from wild *D. melanogaster* faeces, GenBank no. MN684824 [31]), the yeast *Trigonopsis vinaria* (isolated from wild *D. suzukii* ovaries, GenBank #MN684816 [31]), the bacterium *Gluconobacter thailandicus* (isolated from a *Drosophila*-infested grape berry [32]) and the bacterium *Lactobacillus plantarum* (isolated from *D. melanogaster*, GenBank #EU096230 [33]).

To associate flies with these microorganisms (see below), we started each microbial culture from a frozen pure microbial aliquot. The four microorganisms required different culture conditions. We incubated both yeasts at 24°C, 150 r.p.m. in yeast extract–peptone–dextrose (Thermo Scientific™) liquid medium. We incubated the bacterium *G. thailandicus* at 24°C, 150 r.p.m. in Mannitol (MAN, Thermo Scientific™) liquid medium. We incubated the bacterium *L. plantarum* at 30°C, 150 r.p.m. in De Man, Rogosa and Sharpe (MRS, Thermo Scientific™) liquid medium. All four microorganisms were incubated for 12 h; this time, they were allowed to obtain similar cell concentrations in the exponential growth phase (data not shown).

### 2.3. Gnotobiotic flies

We created four groups of gnotobiotic flies for each *Drosophila* species. Each group was associated with a particular bacterium–yeast community, namely *H. uvarum–G. thailandicus*, *H. uvarum–L. plantarum*, *T. vinaria–G. thailandicus* or *T. vinaria–L. plantarum*.

First, we collected eggs on grape juice agar plates presented to mature adults for 12 h and immediately dechorionated them using a method proposed by Koyle and colleagues [34]. We deposited these axenic eggs on a sterile artificial diet (233 g l^−1^ organic banana, 62 g l^−1^ sugar, 62 g l^−1^ inactive dry yeast, 10 g l^−1^ agar; autoclaved at 121°C for 20 min). Three days later, we poured an artificial microbial community, composed of a bacterium (500 colony-forming units (CFUs) suspended in 10 µl freshly sampled from a 12 h culture) and a yeast (*idem*), on the diet.

Once young adults emerged, we separated them per sex and transferred them to a new, sterile artificial diet inoculated with their original microbial community following the procedure described above. We regularly collected flies to control their gnotobiotic status. To this aim, we crushed single adults in sterile phosphate-buffered saline (PBS, Sigma-Aldrich, ref. P4417, 1 tablet/200 ml), for 2 min at 30 Hz with two sterile glass balls (3 mm) using a Tissue Lyser II (Qiagen). We then serially diluted those homogenates in sterile PBS and plated 10 µl drops on four different solid media chosen for their ability to discriminate our strains thanks to their metabolic abilities: MAN, MRS, glucose-based medium (33.3 g l^−1^ ᴅ-glucose, 33.3 g l^−1^ ammonium sulfate, 11.3 g l^−1^ yeast nitrogen base without amino acids (BD Difco™), 15 g l^−1^ agar), and galactose-based medium (33.3 g l^−1^ ᴅ-galactose, 33.3 g l^−1^ ammonium sulfate, 11.3 g l^−1^ yeast nitrogen base without amino acids (BD Difco™), 15 g l^−1^ agar). After an incubation period of 48 h at 24°C (30°C for MRS), we identified microbial colonies on the basis of their morphological properties (colour, transparency, texture and shape), of which we previously controlled the reliability using Sanger sequencing.

### 2.4. Assessing microbial transmission in adult flies

For each *Drosophila* species, we released male–female gnotobiotic pairs in small tubes and observed their sexual behaviour for a few hours. We composed each pair by choosing a male and a female associated with two different microbial communities (see above) with no microbial overlap. We thus released males associated with the microorganisms *H. uvarum* and *G. thailandicus* with females associated with *T. vinaria* and *L. plantarum*, and vice versa. Similarly, we released males associated with the microorganisms *H. uvarum* and *L. plantarum* with females associated with *T. vinaria* and *G. thailandicus*, and vice versa. After observing the presence or absence of mating, we collected and prepared the adults to detect and quantify microbial transmission as described below.

We conducted the experiment over four different mornings, the first hours of the day being favourable to observe high fly mating activity [35]. We inserted a slightly incised blueberry in each experimental tube to provide support, water and food to the flies. Before the experiment, we sterilized the fruit surface using the method of Behar and colleagues [36] and aseptically wounded the berry near the peduncle insertion. We released a total of 127 *D. suzukii* and 175 *D. melanogaster* pairs in small tubes. Copulation time in *Drosophila* lasts at least 25−30 min [35]; we observed each tube every 15 min to ensure the detection of all matings. When a copulating pair separated, we transferred each partner to a separate microtube using a mouth aspirator with sterile end parts. After 3 h, approximately half of the pairs of both species had mated and we collected unmated pairs as described above for mated pairs. Using a Tissue Lyser II (Qiagen), we crushed every adult for 2 min at 30 Hz in sterile PBS with two sterile glass balls (3 mm). We then serially diluted adult homogenates in sterile PBS and plated 10 µl drops of each dilution on the four solid media described above to count the CFUs present in adults. The morphological identification of the four microbial strains enabled us to identify and quantify the microorganisms present in each adult. We used these values to deduce the densities of ‘already present’ symbionts in both adults, i.e. the microorganisms that were associated with the flies before the experiment. We also used these values to detect any microbial transmission between the two adults and to quantify it. Because flies were crushed shortly after their collection, the experiment informs on microbiota transmission events but not on the fate of the transmitted symbionts, which may or may not settle and multiply in their new hosts.

### 2.5. Statistical analyses

We structured our analyses in two sections presented below. We performed both analyses using R 3.6.2 [37]. Backward stepwise selections allowed us to remove non-significant factors from initial full models (*α* = 0.05).

#### 2.5.1. Mating status and microbial transmission

First, we tested whether mating status affected microbial transmission in *Drosophila* pairs. We predicted mating would increase the likelihood of microbial transmission between *Drosophila* males and females. We also expected that the transmission frequency would depend on *Drosophila* species and direction of transmission, i.e. female to male or male to female. To this aim, we built a generalized linear mixed model with binomial distribution using the function glmer from the package lme4 (v1.1-26) [38]. We built the initial model as follows:

— The response variable *‘Microbial transmission’* informs whether or not we detected microbial transmission from one insect of the pair to the other. We defined *‘Microbial transmission’* at the level of every individual: it was coded as ‘1’ if either yeast or bacterium or both were acquired from the conspecific, and coded as ‘0’ if none was acquired.
— The fixed factors were ‘*Mating status*’, i.e. if the pair mated or not, ‘*Fly species*’ and ‘*Direction of transmission*’, i.e. if we tested microbial transmission from the male to the female of the pair or from the female to the male. We also added the two 2-way interactions *‘Mating status’* × *‘Fly species’* and *‘Mating status’* × ‘*Direction of transmission’* as fixed factors.
— We modelled two random factors: ‘*Experimental unit*’, i.e. tube identity, and ‘*Experimental block*’, i.e. experimental day.

#### 2.5.2. Factors affecting microbial transmission in mated flies

Our second approach was restricted to mated individuals and aimed at unveiling the factors that affect transmission between sexual partners. In particular, we hypothesized that some microbial species could transmit more often than others. Microbial cell densities could also influence microbial transmission. We predicted that the more abundant a microorganism was in the donor host, the more likely would be its transmission. We also predicted a negative relationship between the transmission frequency of a microorganism and the microbial load (of other microorganisms) in the recipient host, because microorganisms that colonize a given host would prevent its colonization by newcomers. We tested whether these predictions could differ among *D. melanogaster* and *D. suzukii*, two species with different ecologies and behaviours. To this aim, we built a generalized linear mixed model with binomial distribution using the function glmer from the package lme4 (v1.1-26) [38]. When necessary, we performed multiple contrasts to detect significant differences between factor levels using the package emmeans (v1.6.2-1) with Tukey adjustment [39].

We built the initial model as follows (also summarized in table 1):

— The response variable *‘Microbial transmission’* informs whether or not we detected transmission of a microorganism from a particular kingdom, i.e. yeasts or bacteria, from one fly to the other. We therefore defined *‘Microbial transmission’* at the level of every individual and every microbial kingdom, as we assumed yeasts and bacteria exploit different ecological niches within hosts.
— The fixed factors were ‘*Fly species*’, ‘*Direction of transmission*’ (described in the previous section), *‘Symbiont combination in the recipient and donor hosts’*, i.e. the identity of the microorganisms from a given kingdom associated with the recipient and the donor hosts before the experiment, *‘Number of ‘already present’ microbial cells in the donor host (log transformed)’* , i.e. the number of cells (log transformed) of the focal microorganism associated with the donor host before the experiment, and *‘Number of ‘already present’ microbial cells in the recipient host (log transformed)’*, i.e. the number of cells (log transformed) of the microorganism, of the same kingdom as the focal one, associated with the recipient host before the experiment. We also added the four 2-way interactions between *‘Fly species’* and the other four fixed factors.
— We modelled two random factors: ‘*Experimental unit*’, i.e. tube identity, and ‘*Experimental block*’, i.e. experimental day.

**Table 1.** Statistical model results of the analysis of the transmission of microbiota members between sexual partners as a function of host and symbiont properties. Generalized linear mixed model with binomial distribution and logit link function (*α* = 0.05). *p*-values are coded as *p* < 0.05*, *p* < 0.01**, *p* < 0.001***.

random effects	
*‘experimental unit’*	variance: 0.60, s.d.: 0.78
*‘experimental block’*	variance: 0.15, s.d.: 0.39

fixed effects	
*‘fly species’*	χ² = 0.61, d.f. = 1, *p* = 0.4351
*‘direction of transmission’*	χ² = 0.10, d.f. = 1, *p* = 0.7498
*‘symbiont combination in the recipient and donor hosts’*	χ² = 19.63, d.f. = 3, *p* = 0.0002***
*‘number of ‘already present’ symbiont cells in the recipient host (log transformed)’*	χ² = 2.14, d.f. = 1, *p* = 0.1434
*‘number of ‘already present’ symbiont cells in the donor host (log transformed)’*	χ² = 11.81, d.f. = 1, *p* = 0.0006***
*‘fly species’* × *‘direction of transmission’*	χ² = 7.84, d.f. = 1, *p* = 0.0051**
*‘fly species’* × *‘symbiont combination in the recipient and donor hosts’*	χ² = 8.61, d.f. = 3, *p* = 0.0349*
*‘fly species’* × *‘number of ‘already present’ symbiont cells in the recipient host (log transformed)’*	χ² = 6.70, d.f. = 1, *p* = 0.0096**
*‘fly species’* × *‘number of ‘already present’ symbiont cells in the donor host (log transformed)’*	χ² = 1.44, d.f. = 1, *p* = 0.2297

When microbial transmission was detected, we investigated the influence of the different factors listed above on the number of retrieved microbial cells. To this aim, we built a linear mixed model with log-normal distribution using the function lmer from the package lme4 (v1.1-26) [38]. When necessary, we performed multiple contrasts to detect significant differences between factor levels using the package emmeans (v1.6.2-1) with Tukey adjustment [39]. The variables used in this model (also summarized in electronic supplementary material, table S1) were identical to the ones used in the model described above except for the response variable, named *‘Number of symbiont cells transmitted (log transformed)’*.

In addition to these two models, we used the software JMP (SAS, 18.0.0) to perform ordinary least squares regressions to test the relationship between the response variables *‘Microbial transmission’* and *‘Number of symbiont cells transmitted (log transformed)’* and the factors *‘Number of ‘already present’ microbial cells in the donor host (log transformed)’* and *‘Number of ‘already present’ microbial cells in the recipient host (log transformed)’* for each fly species.

## 3. Results

### 3.1. Mating status and microbial transmission

**Figure 1.**
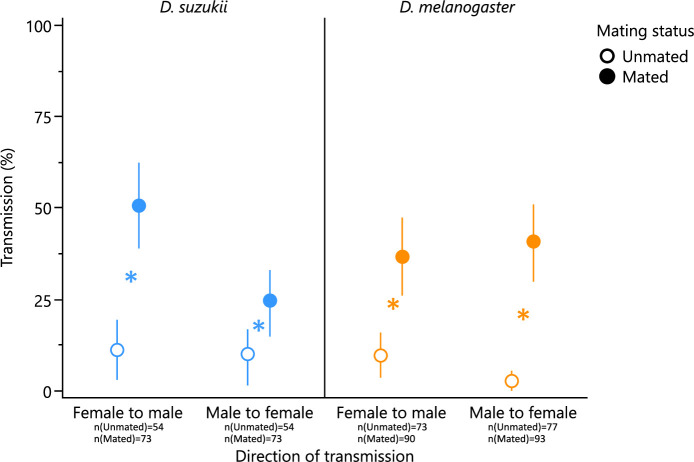
Effect of mating on the transmission frequency of microbial symbionts between *Drosophila* males and females. Symbols and bars indicate mean and 95% CI (calculated using normal approximation method). Asterisks indicate that transmission rates differed significantly between mated and unmated fly pairs (*α* = 0.05).

Mated flies were more susceptible to acquire yeasts and bacteria from their congeners than unmated flies (χ² = 41.23, d.f. = 1, *p* < 0.0001), a phenomenon that did not differ between the two fly species (χ² = 1.87, d.f. = 1, *p* = 0.1714) and between females and males (χ² = 0.29, d.f. = 1, *p* = 0.5870) (figure 1).

### 3.2. Factors affecting microbial transmission in mated flies

**Figure 2.**
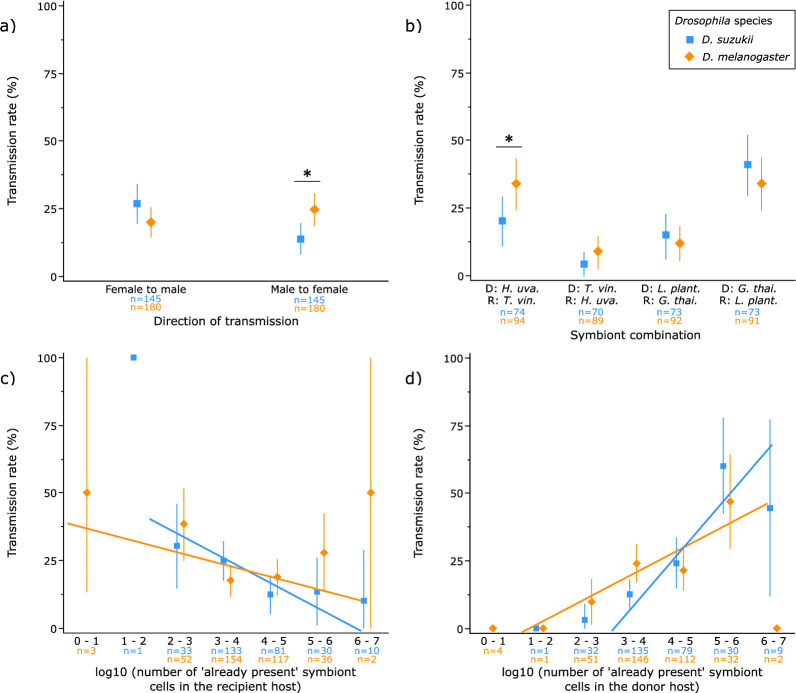
Probability of symbiont transmission between sexual partners as a function of (*a*) the direction of transmission, i.e. female to male or male to female; (*b*) the symbiont combination in donor (D) and recipient (R) hosts analysed per biological kingdom (either yeasts or bacteria: *H. uva.: Hanseniaspora uvarum* (yeast), *T. vin.: Trigonopsis vinaria* (yeast), *L. plant.: Lactobacillus plantarum* (bacterium), *G. thai.: Gluconobacter thailandicus* (bacterium)); (*c*) the number of ‘already present’ microbial cells in the recipient host (log transformed); (*d*) the number of ‘already present’ microbial cells in the donor host (log transformed). For (*c*,*d*), raw *x*-axis data were analysed while *x*-axis data transformed as categorical are presented for graphical convenience. Symbols and bars indicate mean and 95% CI (calculated using normal approximation method). Asterisks indicate a significant difference in transmission rate between two factor levels (contrasts, *α* = 0.05). *n* indicates the number of measurements. For (*c*), the results of the ordinary least squares regression were, for *D. suzukii*, *Y* = 0.5116 − 0.0769 × *X*; *F*_1,286_ = 8.90, *p* = 0.0031; *R*² = 0.03, and for *D. melanogaster*, *Y* = 0.3763 − 0.0396 × *X*; *F*_1,364_ = 2.92, *p* = 0.0881; *R*² = 0.01. For (d), the results of the ordinary least squares regression were, for *D. suzukii*, *Y* = −0.4272 + 0.1591 × *X*; *F*_1,284_ = 41.03, *p* < 0.0001; *R*² = 0.13 and for *D. melanogaster*, *Y* = −0.0664 + 0.0767 × *X*; *F*_1,346_ = 10.51, *p* = 0.0013; *R*² = 0.03.

In mated *Drosophila* pairs, several fly- and symbiont-related factors significantly affected the transmission of yeasts and bacteria (table 1). Females of both species transmitted equally symbionts to their male partners. *D. melanogaster* males however transmitted more often symbionts to females than *D. suzukii* males did (interaction *‘Fly species’* × *‘Direction of transmission’*; χ² = 7.84, d.f. = 1, *p* = 0.0051) (figure 2*a*). Microbial identity in donor and recipient hosts affected transmission frequency with transmission rates varying between 4 and 41% of matings (χ² = 19.63, d.f. = 3, *p* = 0.0002). The yeast *Hanseniaspora uvarum* was more frequently transmitted in *D. melanogaster* than in *D. suzukii* (χ² = 8.61, d.f. = 3, *p* = 0.0349) (figure 2*b*). A greater number of symbiont cells in the recipient host reduced the likelihood of symbiont acquisition in *D. suzukii*, but not in *D. melanogaster* (interaction *‘Fly species’* × *‘Number of ‘already present’ symbiont cells in the recipient host (log transformed)’*; χ² = 6.70, d.f. = 1, *p* = 0.0096) (figure 2*c*). In both species, however, a greater number of symbiont cells in the donor led to a greater likelihood of symbiont transmission between sexual partners (figure 2*d*). If the transmission was successful, the number of transmitted cells was not clearly affected by the combination of symbionts in the donor and recipient hosts, by the number of cells in the donor and recipient hosts, by the host species or by the direction of transmission (electronic supplementary material, 1).

## 4. Discussion

Our experiment revealed frequent transmission of microbiota members between sexual partners in the model insect *D. melanogaster* and the fruit pest *D. suzukii*. Such transmission occurred from males to females and from females to males (figure 2*a*). Microbial identity and cell density in donor and recipient hosts significantly influenced microbiota transmission between sexual partners.

Earlier studies in some *Drosophila* species reported sexual transmission of microorganisms, a phenomenon well established in various insect hosts [3,6–11]. Starmer and colleagues [1] investigated the transmission of various yeasts species during the courtship and mating of the North American fly *Drosophila buzzatii*. Their design quantified symbiont transmission from mono-associated to axenic hosts. Transmission rates appeared high, with recipient hosts positive for donor’s yeast in more than 80% of matings, and were greater than the ones reported in the present study, here from 4 to 41%. The differences may originate from the axenic status of their recipient hosts. We showed that symbiont conditions in recipient hosts can affect the likelihood of symbiont acquisition by sexual partners (figure 2). Another study investigated the transmission of a pathogenic strain of the bacterium *Serratia marcescens* during courtship and mating in *D. melanogaster* [5]. This study also found high transmission rates, precisely 69%, from contaminated males to virgin females. The contaminated males however received a large bacterial dose artificially: donor hosts were inoculated by ‘submerging the posterior quarter of the male’s abdomen […] and genitalia into the broth’ that contained the pathogen. By contrast, our experiment, in which flies associated with microorganisms in their early life in a more ecologically realistic way, showed the importance of symbiont density in the donor host on transmission likelihood (figure 2*d*). Together these observations show that sexual transmission could be frequent in *Drosophila* flies but probably depends on the composition of ‘resident’ microbiota in donor and recipient flies. Moreover, the differences observed between *D. suzukii* and *D. melanogaster* (figure 2*a*–*c*) reveal that the sexual transmission of microbiota members may follow different routes or rely on different processes in different host species.

The microbiota is not a homogeneous community of microorganisms with similar properties. Each member can have its specific metabolism, within-host niche and means of transmission [19,20]. Our experiment exemplified this variability with rates of transmission varying by a 10-fold factor between combinations of symbionts in donor and recipient hosts (figure 2*b*). This observation echoes research on the establishment of commensal bacteria in the gut of *D. melanogaster* adults where colonization likelihood depends on the composition of the ‘already present’ community [40,41]. Our work however does not allow us to discriminate the effect of a particular strain in the donor host from that of another one in the recipient. As we tested two strains of yeasts and two strains of bacteria, if one strain was in the donor, the other had to be in the recipient. In addition, we did not identify the routes and mechanisms of sexual transmission at play in our experiment. In *D. buzzatii*, yeasts sexually acquired by mated females are found in their abdomen. By contrast, yeasts sexually acquired by mated males are found in their heads [1]. This suggests microorganisms transferred during copulation may follow either external routes, e.g. because they are present on insect cuticle, and internal routes, e.g. because they are present in reproductive organs or fluids such as *Trigonopsis vinaria* sampled from *D. melanogaster* ovaries, or can even be acquired after copulation through ingestion via fly grooming. Moreover, our study reports the occurrence of acquired symbionts shortly after mating. It is therefore probable that a portion of the microbial transmission events we observed would not lead to a stable microbial colonization of the host. Further work will be necessary to unveil the mechanisms of microbiota sexual transmission in *Drosophila* flies.

Considering the consequences of microbial transfers between sexual partners might be important for insect pest control, in particular for the SIT where numerous sterile insects released end up mating with wild counterparts [42]. Symbionts that would negatively affect hosts, ideally females, and that would be frequently transmitted during courtship or mating could be used to improve SIT efficacy. In that case, sterile males should be inoculated with a microbial agent prior to their release in the field. This strategy called boosted SIT is the topic of current research in several plant pest and disease-vector insects [43–46]. Sexual transmission of microbiota members may also be used to reduce direct damages to crops. For example, it is well established that the microorganisms associated with *D. suzukii* larvae, partly acquired from their mothers [20], can trigger sour rot, a fruit disease responsible for crop losses [25]. Inoculating mass-reared flies with microbial symbionts chosen for their antagonistic effect on fruit-degrading microorganisms [47] might help reduce crop damages when females lay viable eggs. Understanding the field transmission of pest-associated symbionts is hence a potent tool for agroecological crop protection. Sexually transmitted symbionts can also be a problem for SIT-based pest control. The microbiota communities of wild and captive insects can be very different [48,49]. This is particularly true in *Drosophila* flies; their microbiota is very flexible and mostly depends on context [50,51]. Mass-reared insects released in the field may become the vectors of microorganisms absent or rare in ecosystems and agrosystems. Sexual transmission could facilitate the spread of these microorganisms to wild host populations and therefore constitute an overlooked non-target effect of the SIT [52].

## Data Availability

Data are available within the Zenodo repository [53]. Electronic supplementary material is available online [54].
